# Physiology-Based Revascularization of Left Main Coronary Artery Disease

**DOI:** 10.1155/2021/4218769

**Published:** 2021-02-10

**Authors:** Peter Kayaert, Mathieu Coeman, Sofie Gevaert, Michel De Pauw, Steven Haine

**Affiliations:** ^1^Department of Cardiology, Ghent University Hospital, Ghent, Belgium; ^2^Department of Cardiology, Jan Yperman Ziekenhuis, Ypres, Belgium; ^3^Department of Cardiology, Antwerp University Hospital, Antwerp, Belgium; ^4^Department of Cardiovascular Diseases, University of Antwerp, Antwerp, Belgium

## Abstract

It is of critical importance to correctly assess the significance of a left main lesion. Underestimation of significance beholds the risk of inappropriate deferral of revascularization, whereas overestimation may trigger major but unnecessary interventions. This article addresses the invasive physiological assessment of left main disease and its role in deciding upon revascularization. It mainly focuses on the available evidence for fractional flow reserve and instantaneous wave-free ratio, their interpretation, and limitations. We also discuss alternative invasive physiological indices and imaging, as well as the link between physiology, ischemia, and prognosis.

## 1. Introduction: Left Main Disease as the Pinnacle of Coronary Artery Disease

Significant left main (LM) coronary artery stenosis is found in at least 7% of patients undergoing coronary angiography (CA) [1]. In the era when only medical treatment (MT) was available, patients with significant LM disease had a particularly poor prognosis with mortality rates as high as 43% at 5 years [2, 3]. Nowadays, myocardial revascularization (MR) by coronary artery bypass grafting (CABG) or percutaneous coronary intervention (PCI) is performed to relieve ischemic symptoms and/or to improve prognosis [4]. It is more effective than MT alone in reducing ischemia and angina, and when the ischemic burden is large, it may improve long-term survival [3, 5–9]. Given that the perfusion territory of the LM extends up to 84% of the left ventricle, MR is particularly important in significant LM disease [3, 7–11]. Even after state-of-the-art percutaneous or surgical MR, patients may still experience major adverse events. In the recent EXCEL trial, the event rate of a composite of death, stroke, or myocardial infarction (MI) at 5 years was around 20% (22.0% in the PCI group and 19.2% in the CABG group; *P*=0.13) [12]. Considering the poor outcome of the patients with significant LM lesions which are not revascularized and the event rates in the revascularized patients, it is of critical importance to correctly assess the significance of a LM lesion. Underestimation of significance carries the risk of inappropriate deferral of MR, whereas overestimation may trigger major but unnecessary interventions. This article addresses the invasive physiological assessment of LM disease and its role in deciding upon revascularization.

## 2. Estimation of Significance: Physiology to the Rescue

Most of the evidence in management of LM disease has been gathered using a significance threshold of 50% luminal diameter stenosis (DS) as determined by visual assessment of two-dimensional X-ray CA [13–15]. This method has two fundamental limitations.

First, X-ray CA only provides silhouette images of the injected contrast dye and therefore represents only a luminogram. Atherosclerosis is often a diffuse disease and luminal narrowing may be absent or minimal due to positive remodeling (Glagov phenomenon) [16]. It can therefore be difficult to define completely normal segments and reliably determine a DS grade (ratio of minimal lumen diameter of a lesion to the diameter of an adjacent normal segment). Underestimation of disease in a “normal” reference vessel may lead to underestimation of lesion severity. LM lesions are particularly challenging to assess by classical two-dimensional CA and even by two- and three-dimensional quantitative CA (2D and 3D QCA) [17]. A healthy reference segment within the LM is often lacking. To avoid underestimation, one should therefore always keep Murray's branching law in mind. The LM should at least have a larger reference diameter than its largest daughter branch. If this is not the case, LM disease is very likely present [18]. More importantly, lesion angulation and eccentricity, reverse tapering, bifurcation anatomy, overlapping branches, foreshortening, ostial LM catheter-induced distortion, or pseudostenosis from catheter tenting may hamper LM assessment [13, 19–21]. Interobserver variability is high, and correlation with measurements by intravascular ultrasound (IVUS) is poor [21, 22]. When compared to a postmortem histological examination, LM lesions are more often underestimated by CA, though overestimation can occur, as in underfilling or spasm of the LM [23].

Second, a lesion may be visually important, but not functionally significant and vice versa. This visual-functional mismatch occurs not only because of the limitations of CA in reflecting the epicardial conduit resistance, but also because of the influence of the myocardial area and mass supplied by the specific artery [24, 25]. A pressure drop across a stenosis is due to both friction losses along the lesion entrance and throat (Poiseuille's law) and separation losses due to convective acceleration along the narrowed section (Bernoulli's law). The relationship between pressure gradient (Δ*P*) and flow velocity (*v*) is described by the quadratic equation Δ*P* = *Av* + *Bv*^2^, where the first and second terms, respectively, represent friction and separation losses. The pressure drop and thus the flow-limiting behavior of a coronary stenosis are largely caused by the inertial exit losses that scale with the square of the flow, which implies that flow is a major determinant in the equation [26, 27]. Coronary blood flow is related to the size of the subtended viable myocardial bed. Therefore, flow volumes are higher in the vessels supplying larger areas of myocardium and especially in their proximal parts, explaining the higher pressure drops in proximally located lesions such as the LM [24, 28]. This explains why a LM lesion may cause a significant pressure drop and thus be functionally significant even when it appears relatively mild on CA. Importantly, flow through a stenosis is also dependent on any resistance caused by up- and/or downstream lesions, if present, and on the resistance of the microcirculation and its autoregulatory reserve [26, 29].

Overall, estimating lesion significance only by assessing the degree of stenosis on CA is not sufficiently accurate, but due to the aforementioned reasons, this is particularly true for a lesion located in the LM [30]. In a study involving 152 LM lesions, almost one out of two LM lesions (versus one out of three non-LM lesions) would have ended up being misclassified when the classical 50% DS angiographical cut-off was used instead of a fractional flow reserve (FFR) ≤0.80 as a measure of functional significance [30]. Underestimation of LM lesion significance is particularly more common (sensitivity of 50% DS for an FFR ≤0.80 was only 35%) [30]. Current European guidelines therefore advocate performing additional invasive physiological assessments by FFR or by instantaneous wave-free ratio (iFR) to assess the hemodynamic relevance of every intermediate-grade stenosis in which lesion-related ischemia is not yet documented (Class I, level of evidence A guideline) [4]. In LM lesions, this evaluation is indicated in a stenosis of more than 50%, but less than or equal to 90% (Class I, level of evidence A) [4]. A LM lesion of more than 90% is considered an indication for revascularization without further proof of ischemia [4].

## 3. Physiology-Based LM Revascularization: Evidence, Limitations, and Pitfalls

### 3.1. FFR

#### 3.1.1. Rationale and Evidence

FFR refers to the maximal myocardial blood flow in the presence of coronary stenosis (*Q*_S_), as a fraction of the maximal flow that theoretically would be achievable in the absence of the stenosis (*Q*_N_) [26]. It was introduced in 1993 as an index that could be estimated by the ratio of the mean distal coronary pressure (*P*_*d*_) to the mean aortic pressure (*P*_*a*_) during maximal dilation of the coronary vascular bed, a state called maximal hyperemia [26]. The *P*_*a*_ and *P*_*d*_ are simultaneously measured by a pressure transducer connected to the guiding catheter and a pressure sensor-equipped guidewire, respectively [31]. To correctly predict flow by measuring pressures, Pijls et al. assumed a proportional linear relationship between coronary perfusion pressure and flow during maximal hyperemia.

The advent of the pressure-derived FFR dramatically boosted the use of physiology in clinical practice, as direct measurement of coronary flow and coronary flow reserve (CFR) never got widely used in clinical practice due to being nonspecific to the epicardial circulation and technically challenging. The research group of Pijls and De Bruyne was subsequently able to show that one can safely defer treatment of lesions with an FFR >0.75 (DEFER trial), that adding FFR to angiography for guidance of revascularization results in significantly less major adverse cardiac events, MACE (FAME study), and that FFR-guided PCI improves outcome as compared to MT alone (FAME 2 study). These three landmark randomized trials together with a patient-level meta-analysis and a bulk of observational data led to the Class IA indication for use of FFR in guiding revascularization [4, 32–35].

LM disease was however extremely rare in the early validation studies of FFR, and the Class IA recommendation for measurement in LM lesions in the European guidelines is solely based on limited observational data (Table 1). A non-patient-level meta-analysis of six prospective cohort studies involving 525 patients indicated similar MACE rates (a composite of all-cause mortality, MI, and subsequent revascularization) in patients undergoing CABG for a pathological/positive FFR (FFR+) as in patients with a negative FFR (FFR-) who were deferred for CABG and only treated medically [36]. Deferred patients did have a significantly higher MR rate during follow-up [36]. Although in favor of FFR in LM stenosis, the meta-analysis is underpowered for hard clinical endpoints, such as mortality and MI, and limited as well by the nonuniform FFR threshold for a “positive FFR.” Overall, the 0.75 cut-off (<0.75 considered as FFR+) was mostly used, but in the largest and most recent cohort from Hamilos et al., 0.80 (<0.80 considered as FFR+) was applied as cut-off [36, 41, 42]. The studies may also have suffered from selection bias and differences in local practice. For example, in Hamilos' study, it was allowed to measure the LM FFR proximal to visible downstream stenosis, which unavoidably must have resulted in different FFR values from those when it would have been measured distal to the downstream stenosis. Recently, the 2-year outcomes of 1447 FFR-deferred lesions in the J-CONFIRM study were published [44]. A LM as target lesion (*N* = 37) was an independent risk factor of target vessel failure during follow-up (HR 5.89; 95% CI: 2.72–12.8; *P* < 0.001).

Although FFR is mostly accurate and clinically very useful, it is important to understand its fundamental limitations and the challenges related to the assessment of LM disease.

#### 3.1.2. Limitations

FFR assumes maximal hyperemia to be present, meaning that resistance to flow should be minimized at both the epicardial and microcirculatory level of the coronary circulation. This is achieved by intracoronary (IC) administration of nitrates and IC or intravenous (IV) administration of adenosine (or alternatively papaverine or regadenoson), respectively. As flow is dependent on resistance by up- and/or downstream lesions and on the resistance of the microcirculation, flow acceleration during hyperemia and resulting pressure gradients and FFR values are as well.

*(1) Influence of Downstream Disease*. Up to 72% of patients with LM disease have diffuse or distal LM disease, implying a possibly different impact on flow in both branches [41]. Therefore, the FFR of a LM stenosis is not necessarily identical, whether measured in LAD or LCx, even when resistance distal to the LM stenosis is assumed to be minimal [20]. More importantly, only a minority has isolated LM disease as downstream disease is common: LAD and LCx disease were present in 44% and 33%, respectively, in the largest registry [42]. Downstream lesions and the LM lesion then act as serial lesions and under conditions of maximal hyperemia, even intermediate lesions can influence each other as the maximal attainable flow velocities will be less than those in the absence of a second stenosis [20, 45]. Lower hyperemic flow velocities will lead to lower pressure gradients, which translate into higher FFR values and underestimation of the hemodynamic significance of the LM lesion.

In serial lesions, it is feasible to calculate the FFR of each lesion individually by a complex but validated formula. The procedure requires measuring the coronary wedge pressure, a diagnostic procedure that may result in vessel trauma [46, 47]. A safer and more practical strategy of treating the lesion that causes the most significant pressure step-up upon a manual FFR pullback resulted in a good short-term outcome in small series [48, 49]. One still has to consider that a stable hyperemic phase is necessary to allow for reliable pressure measurements during pullback, something that even with IV adenosine is not achievable in up to 43% of cases [50]. Repeating the FFR measurement after stenting is also an essential part of that strategy as relieving the resistance to flow by the treatment of one lesion increases the flow and thus the pressure gradient over the residual lesion(s) by an unpredictable amount [27, 47, 51]. The resulting FFR may therefore still be below 0.80 in some patients, implying that the residual lesion was hemodynamically significant from the beginning [27]. In one series, only 53% of patients ended up having only the lesion that caused the largest pressure step-up treated [48]. This is particularly relevant in LM disease as one can end up treating the accompanying non-LM disease, assuming it to be significant from the FFR pullback, and still find an FFR<0.80 due to the residual LM lesion. If this could have been known upfront, potentially a more appropriate percutaneous or surgical revascularization strategy would have been chosen.

Recognizing the presence of downstream disease is crucial but may be very challenging due to the visual-functional mismatch. In the recent DEFINE PCI study, 24% of patients were still ischemic after PCI due to unrecognized focal (81.6%) or diffuse (18.4%) disease [52]. In practice, one should strongly consider performing pressure measurements towards both LAD and LCx, even when the LM disease appears isolated [20].

**Figure 1:**
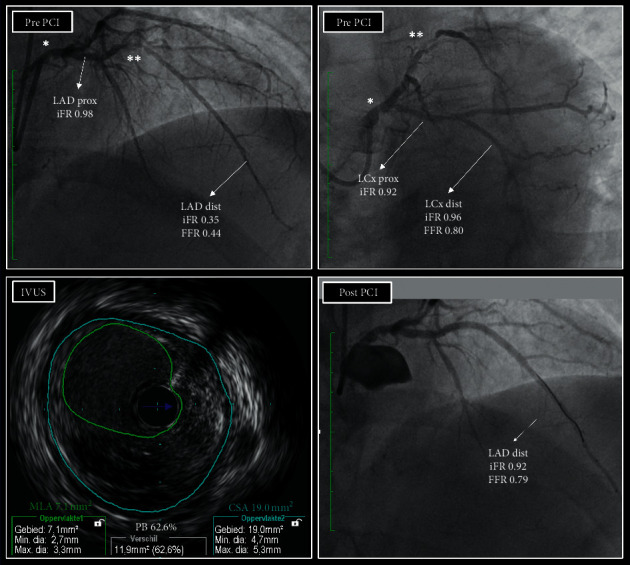
A practical example of a LM lesion with downstream disease. A patient with effort angina had a LM lesion (^*∗*^) which appeared angiographically at least moderate, but also a severe lesion at the level of the mid LAD (^*∗∗*^). The LCx showed some disease but was clearly the least affected daughter branch of the LM. iFR distal to LAD was 0.35, indicating flow-limiting disease upstream, but assessment of the iFR along the course of the LAD revealed that the drop in iFR was mainly caused by the mid LAD lesion. In the proximal part of the LAD, the iFR was 0.92, suggesting that the LM lesion was not physiologically significant. iFR in the LCx was 0.92 in the proximal part and 0.96 in the distal part, which seemed to confirm this. However, since the disease in the LAD was so severe that it may also have been flow-limiting at rest, stable resting conditions may have been absent and, theoretically, the iFR might have been lower at that level if the downstream disease was not so critical. FFR towards LAD was 0.44, also indicating critical disease upstream. FFR towards LCx was 0.80, borderline significant. Since the disease in LAD was severe and the value was below 0.85, the LM lesion might still have been flow-limiting. Given the iFR and FFR findings, the operators decided to also evaluate the LM lesion with IVUS. The MLA was 7.1 mm^2^ (above 6.0 mm^2^), and the operators decided to defer the LM, treat the LAD lesion, and repeat physiological assessment. After PCI, the result was good by IVUS, and the iFR was 0.92. FFR was 0.79, just below 0.80 but above 0.75. Maximal preventive treatment was installed and, 20 months later, the patient is still asymptomatic and event free. Because of the disease in the LM (plaque burden, PB, on IVUS was 62%) and the final FFR value, the operators did plan a follow-up angiography with physiological assessment and IVUS 2 years after the index procedure. LM: left main; LAD: left anterior descending artery; LCx: left circumflex artery; iFR: instantaneous wave-free ratio; FFR: fractional flow reserve; IVUS: intravascular ultrasound; MLA: minimal lumen area; CSA: external elastic membrane cross sectional area; PB: plaque burden; PCI: percutaneous coronary intervention. Of note, the higher iFR value in the distal LCx versus in the proximal LCx can be explained by the fact that the distal part of the LCx is located significantly lower than the aortic root and thus exposed to a higher hydrostatic pressure.

In the unique setting of a LM lesion accompanied by downstream disease in only one daughter vessel, it is considered best practice to measure the FFR of the LM lesion by placing the pressure wire down the nondiseased daughter vessel. Carefully designed theoretical, in vitro, in vivo, and human studies show that the FFR value, as measured in the nondiseased daughter vessel, is significantly higher than the true LM FFR. However, the downstream disease has to be very severe (FFR < 0.60 in the diseased daughter vessel up to a chronic total occlusion, CTO) to falsely elevate the FFR result beyond the cut-off point of 0.80 and thus underestimate the LM lesion severity. In the presence of severe downstream disease, a 0.85 FFR cut-off for the LM has been proposed (≤0.85 considered as FFR+), as with a 0.80 cut-off, the true FFR of the LM could occasionally end up being ≤ 0.75, and with an FFR value measured >0.85, this would never happen [27, 50, 53–56]. A cut-off for FFR in the disease-free daughter vessel to exclude a true LM FFR ≤0.80 has not been proposed though. Therefore, experts suggest the use of IVUS and a minimal lumen area (MLA) >6 mm^2^ to rule out significant LM disease if the FFR towards the diseased branch is <0.60, or more pragmatically in any case where downstream disease in LAD or a large LCx appears severe (even if only ostial) and its upfront treatment is not considered an option [13, 57] (Figure 1).

Occasionally, a LM lesion may be accompanied by a CTO of the right coronary artery (RCA) receiving collaterals from the left coronary artery (LCA). The perfusion territory of the LM therefore enlarges by the collateralized territory distal to the CTO, especially when the latter is still viable (myocardial blood flow to nonviable myocardium is very low) and contracting [58]. One could expect that the measured LM FFR value will be lower than that in the absence of a collateralized CTO. It was shown that recanalization of a CTO generally increases FFR of the predominant collateral donor vessel [59, 60]. At four months, 18% of the initially FFR+donor lesions had become FFR in one study [60]. Therefore, to correctly evaluate the functional significance of a LM lesion, one has to treat the CTO in the RCA first or evaluate the LM severity differently. In the catheterization laboratory, this can be done by IVUS [13].

*(2) Influence of the Microcirculation*. The functional status of the coronary microvasculature is a major determinant of the response of coronary flow to a hyperemic stimulus. The minimal microvascular resistance during maximal hyperemia (HMR) is highly variable [61]. A lower minimal microvascular resistance will lead to a higher flow acceleration and result in a larger pressure drop and thus lower FFR value, and vice versa [26].

A particular situation is that of an acute coronary syndrome (ACS). If the LM is the culprit lesion or the culprit lesion is situated in the left system, the use of FFR to assess an intermediate LM lesion should be avoided [62]. FFR can be used if the culprit lesion is in the RCA, meaning that the LM lesion is likely a nonculprit lesion. The microcirculatory vasodilatation induced by a hyperemic stimulus may still be transiently blunted in the acute phase of an ACS, affecting also myocardial territories remote to those subtended by a nonculprit stenosis [63, 64]. Patients that have a nonculprit lesion deferred based on physiological assessments have consistently higher MACE rates than patients with deferred stable lesions [64, 65]. Although multiple factors likely contribute to this, the fact that MACE is largely driven by coronary revascularization suggests that, in the setting of ACS, pressure-based indices may not be able to accurately select those lesions that can be safely deferred [64].

*(3) Influence of RA Pressure.* The originally validated equation to calculate FFR included the central venous pressure, *P*_*v*_ (“myocardial” FFR=(*P*_*d*_ − *P*_*v*_)/(*P*_*a*_ − *P*_*v*_) at maximal hyperemia), as measured by a catheter positioned in the right atrium [31]. For practical reasons and since *P*_*v*_ is mostly low, the *P*_*v*_ is generally neglected and the simplified ratio, (*P*_*d*_/*P*_*a*_), is used. However, inclusion of *P*_*v*_ lowers the resulting FFR value. Toth et al. found in a large cohort that, in 9% of cases, FFR values >0.80 corresponded with corrected myocardial FFR values of ≤0.80 (though never ≤0.75) [66]. In another cohort, it was shown that, within a normal range right atrial pressures (<8 mm Hg), FFR values up to 0.83 may correspond to corrected myocardial FFR values of ≤0.80 [67]. In the case of atrial pressures of 15 mm Hg and higher, this can even happen with FFR values up to 0.85, especially if systolic blood pressure is low [67]. Not taking the *P*_*v*_ into account may therefore result in misclassification, even when one would still hold on to a 0.75 cut-off [66–68]. FFR is meanwhile clinically well validated using the simplified formula and the 0.80 cut-off, but one should consider the effect of *P*_*v*_ especially when confronted with particularly high right atrial pressures and/or FFR values around the cut-off. As significant LM disease may result in left ventricular impairment and heart failure, *P*_*v*_ values may be elevated, and it may be of interest to measure them and calculate the myocardial FFR.

### 3.2. iFR

#### 3.2.1. Rationale and Evidence

In 2012, the group of Davies developed a hyperemia-independent pressure-derived index of stenosis severity, the instantaneous wave-free ratio, iFR [69]. By performing wave intensity analysis (WIA) in 39 stenoses, the investigators found a period, beginning 25% of the way into diastole and ending 5 milliseconds before the end of it, in which wave intensity and microcirculatory-originating pressure returned to zero. Within this wave-free period (WFP), intracoronary resistance was very low but foremost particularly stable so that intracoronary pressure and flow were seen to decline together linearly. iFR is measured as the (*P*_*d*_/*P*_*a*_) ratio during the WFP. The investigators showed that this pressure-based index was closely correlated to FFR in 157 lesions [69].

Under resting conditions, increasing stenosis severity will progressively lead to a decrease in the coronary pressure distal to the stenosis as the pressure gradient across the stenosis will rise. This is not only due to the increasing resistance to flow within the lesion but also a consequence of the decrease of microvascular resistance related to the adaptive autoregulatory vasodilation of the microcirculation [70]. A decreasing (*P*_*d*_/*P*_*a*_) or a lower iFR thus means that the lesion itself has become more severe and/or the autoregulatory reserve has progressively been exhausted. Therefore, one can state that iFR measures the physiological impact of a coronary stenosis on the distal coronary bed [71, 72].

The diagnostic accuracy of iFR in detecting ischemia was tested against FFR and other invasive and noninvasive parameters of ischemia and found to be comparable [71, 72]. Of note, iFR appeared to closely agree with CFR, as derived by PET [73]. iFR was even more closely related to CFR (measured invasively) than FFR [74]. Subsequently, 2 large randomized outcome trials were performed. DEFINE-FLAIR and iFR-SWEDEHEART randomized 4529 patients in total to a MR strategy (treatment by PCI or deferral of PCI) based on iFR (≤0.89 considered as iFR+ and indication for treatment, >0.89 considered as iFR- and an indication for deferral) versus one based on FFR (≤0.80 considered as FFR+ and an indication for treatment, and >0.80 considered as FFR- and an indication for deferral). The cardiovascular outcome of an iFR-based deferral or revascularization proved to be noninferior to the strategy based on FFR in both trials for up to two years (for DEFINE-FLAIR and iFR-SWEDEHEART, the 2-year results were presented by Justin Davies at TCT 2019 and Ole Fröbert at TCT 2018, respectively; the pooled patients analysis of both studies was presented by Javier Escaned at the 2020 PCR e-Course) [75, 76]. These results led to the Class IA indication for iFR to guide MR [4].

This recommendation was extended to LM lesions of more than 50%, but less than or equal to 90% if no prior proof of ischemia has been obtained [4]. However, even more so than with FFR, specific outcome data in LM lesions are very limited (Table 1). LM lesions were excluded from DEFINE-FLAIR and were extremely rare in iFR-SWEDEHEART [75, 76]. In 2020, the results of the partially prospective and partially retrospective DEFINE-Left Main Registry were reported [43]. 314 patients with an intermediate LM lesion were treated or deferred for treatment based on an iFR ≤ or >0.89, respectively. Over a median follow-up of 30 months, no significant difference was found in MACE. Unfortunately, in 100 other patients (24.2% of the initially enrolled 414 patients), the operator decided to override the guidance by the iFR result, and in the 26 (26%) subsequently revascularized patients (despite an iFR > 0.89), MACE occurred numerically less frequently than in the 74 (74%) deferred patients (despite an iFR ≤ 0.89). The difference was however not statistically significant.

iFR measurement was better tolerated in DEFINE-FLAIR and about 10% faster and cheaper. A particular advantage of iFR in the subset of LM may be that it is likely less dependent on lesion interplay, since under resting conditions flow is stable across all but the most critical lesions [70, 71, 75, 77]. In contrast to FFR, which needs time-averaging over several cardiac cycles to ensure constant and minimal intracoronary resistance, iFR is measured on a beat-by-beat basis which allows for mapping the iFR values along the entire coronary artery and across several stenoses [69, 71]. If downstream disease is angiographically very severe or extensive, one should consider the possibility that it is flow-limiting at rest and causing interplay with a LM lesion. Therefore, if in such a case the iFR distal to the LM lesion is borderline negative, we would suggest assessing the LM lesion additionally with intracoronary imaging to confirm that the LM MLA is indeed larger than 6 mm^2^ (Figure 1).

Coregistration of the physiological map with the angiogram has been made possible by combining iFR values obtained during a manual pullback with real-time computer tracking [78–81]. This technology helps not only to discriminate the importance of several consecutive lesions but also to detect diffuse disease [71]. iFR coregistration technology also allows virtual stenting as the estimated iFR after virtually removing a stenosis on the pullback trace accurately predicted the post-PCI iFR in several series [79–81].

#### 3.2.2. Limitations

iFR had a good diagnostic performance when compared to the FFR in a dedicated cohort of LM lesions, but disagreement between iFR and FFR on the functional significance of the stenosis did occur in 19% of the 91 cases included in this study [82].

*(1) Discordance between iFR and FFR*. Discordance between iFR and FFR has previously been reported in up to 20% of mainly non-LM lesions. The disparity is likely due to differences in hyperemic flow velocities [74, 83–85].

It was shown that *iFR−/FFR+ patients* generally have a preserved CFR, comparable to the CFR in iFR−/FFR− patients (high), while iFR+/FFR− patientsgenerally have a low CFR [74, 83–85]. In the iFR−/FFR+ group, the myocardial flow is comparable to that in nonobstructed vessels, likely attributable to a relatively healthy microcirculation that provides a strong adaptive autoregulatory response at rest (unexhausted vasodilatory reserve). This healthy microcirculation can lower its resistance substantially following a hyperemic stimulus, thus increasing flow velocities and pressure gradients over the lesion (lower FFR values). In vessels supplying a very large myocardial mass, like the LM and the proximal LAD, the change in coronary flow from rest to maximal hyperemia is greater [86]. Therefore, iFR/FFR discordance may theoretically be more common in LM lesions. Some observational data do suggest that iFR−/FFR+ occurs more often in LM and proximal left anterior descending artery (LAD) lesions [86–88].

In *iFR+/FFR− patients*, the hyperemic flow is more blunted (low CFR), likely due to downstream diffuse disease and/or a microcirculatory disease limiting adenosine-mediated vasodilation (exhausted vasodilatory reserve). The flow is less accelerated upon hyperemia, resulting in smaller pressure gradients (higher FFR values). The CFR in iFR+/FFR−patients is comparable to the CFR in iFR+/FFR+ patients (low) [74, 83–85]. Some found iFR+/FFR− to be more common in diabetes, but data are limited [89, 90]. Some data suggest that it is also more common among older patients [88, 90]. This may be related to the recent finding that aging is associated with a progressive increase in minimal microvascular resistance and a progressive decrease in adenosine-induced hyperemic flow in both obstructed and nonobstructed coronary arteries while resting flow remains more stable [91]. This results in increasing FFR values with age, while iFR remains relatively stable (data presented by Javier Escaned at the 2020 PCR e-Course) [92]. In the DEFINE-FLAIR and iFR-SWEDEHEART studies, younger patients (<60 years old) more often (12% more) underwent MR if they were randomized to an FFR-based strategy than if they were randomized to an iFR-based strategy [92]. There was also a statistical trend that age affected MACE in the patients deferred on FFR (adjusted HR: 1.95; 95% CI: 1.03 to 3.70; *P*=0.06) [92].

As mentioned, FFR as it is currently measured does not incorporate the central venous pressure. Theoretically, the input of this value into the equation may also influence the amount of iFR/FFR discordance. Interestingly, in the single dedicated LM study where both iFR and FFR were measured, the LVEF was an independent predictor of iFR/FFR discrepancy with larger iFR-FFR differences found for lower LVEF values [82]. It could be hypothesized that the low LVEF patients may have had higher central venous pressures and/or more microvascular dysfunction which could have led to higher FFR values.

Because of the relatively high prevalence of iFR/FFR discordance, possibly even higher in LM lesions, following the iFR result instead of the FFR result has a major impact on the decision as to whether or not to revascularize patients. In the Syntax II study, for example, both iFR and FFR assessment were performed in 310 lesions (mainly in the lesions with an iFR 0.86–0.93). If the iFR result (with the 0.89 cut-off) would have been used to decide upon lesion significance instead of FFR, 44% of the lesions would have been reclassified.

Long-term clinical outcome data of patients (regardless of LM involvement) with discordant iFR/FFR results are very limited. Data from the FFR-FRIENDS study suggest good outcomes in deferred patients [93]. Over 80% of iFR−/FFR+ lesions have an FFR between 0.75 and 0.80, and in this FFR zone (initially often referred to as the grey zone), the net benefit on MACE of PCI over MT is limited [94–96]. A pooled patient-level meta-analysis of DEFINE-FLAIR and iFR-SWEDEHEART indicates that, even though in the iFR group patients were 5% less often revascularized, the MACE rates of the iFR-deferred patients were similar to those of the FFR-deferred patients up to two years [71, 92]. Moreover, a post hoc analysis of the 872 patients in DEFINE-FLAIR that had LAD lesions deferred shows no higher MACE rates with iFR vs. FFR (even significantly less: 2.44% vs. 5.26%; adjusted HR: 0.46; 95% CI: 0.22 to 0.95; *P*=0.04) [97].

With regard to the special situation of an RCA CTO receiving collaterals from the left system and the ACS setting, the same limitations overall apply to iFR as to FFR, with some indication that iFR may be better suited than FFR to decide upon deferral of nonculprit lesions in ACS as those patients had numerically less unplanned revascularization during follow-up than the FFR-deferred lesions [60, 64].

### 3.3. Practical Challenges with FFR and iFR

A meticulous technique is required for physiological measurements to be correct and has been extensively described elsewhere [98]. As LM disease may involve the ostium or proximal part of the LM (to an extend sometimes difficult to assess angiographically), engaging the guiding catheter may obstruct flow into the LM causing damping/wedging of the recorded aortic pressure, resulting in a false lower *P*_*a*_ value and a false higher (*P*_*d*_/*P*_*a*_) value. For pressure equalization and measurements, the guide catheter should therefore be disengaged. This may be troublesome. Guide catheters with side holes are no solution since the pressure measured by such a guide catheter is a composite of the pressure at the tip and at the side holes. To stabilize a disengaged regular guide catheter, one may consider downstream advancing an additional coronary wire as a buddy wire [20]. IC administration of adenosine without spilling is impossible with a disengaged guide catheter, which is why IV administration is recommended (the latter is also necessary for FFR pullbacks). To exclude false pressure gradients due to signal drift, the signal stability has to be checked after measurements. Although iFR measures the (*P*_*d*_/*P*_*a*_) ratio only within the WFP and therefore the values are more spread (higher dynamic range) than (*P*_*d*_/*P*_*a*_) ratio values (being averages over the entire cardiac cycle), iFR still has a lower dynamic range than FFR. Therefore, with iFR, particular attention is needed to signal drift (more than 0.02 units should not be accepted) as this may lead to misclassification of lesions and misinterpretation of pullback traces [99].

An overview of the strengths and weaknesses of FFR and iFR in the setting of LM is represented in Table 2.

### 3.4. Alternatives to FFR and iFR

#### 3.4.1. Alternative Resting Indices

The development of iFR and the available outcome data renewed the interest in resting pressure indices other than the resting whole-cycle (*P*_*d*_/*P*_*a*_) (the average (*P*_*d*_/*P*_*a*_) during the entire cardiac cycle). These so-called nonhyperemic translesional pressure ratios (NHPRs) are the “diastolic pressure ratio” (DPR) (Opsens, ACIST), defined as the average (*P*_*d*_/*P*_*a*_) during the entire diastole); another “diastolic pressure ratio” (dPR) (Erasmus MC, Rotterdam), defined as the (*P*_*d*_/*P*_*a*_) during the “flat” period of the d*P*/d*t* signal); the “diastolic hyperemia-free ratio” (DFR (Boston Scientific), defined as the average (*P*_*d*_/*P*_*a*_) during the period between *P*_*a*_ < mean *P*_*a*_ and downsloping *P*_*a*_); and the “resting full-cycle ratio” (RFR (Abbott, Coroventis), defined as the lowest mean (*P*_*d*_/*P*_*a*_) ratio during the entire cardiac cycle). The latter is not strictly a diastolic index though as this lowest (*P*_*d*_/*P*_*a*_) ratio was detected in systole in 12.2% of all examined cardiac cycles in its original validation study [100]. Numerically, the indices are tightly correlated, as demonstrated by comparison with former observational registries, and for clinical use, most experts consider them as equivalent [101]. Follow-up of deferred patients from the IRIS-FFR registry, the 3V FFR-FRIENDS study, and the 13N-ammonia PET registry did not show any difference in prognostic performance [102, 103]. Coregistration and virtual stenting are currently limited to iFR.

#### 3.4.2. Angiography-Based FFR

Technology has been developed to calculate FFR based on 3D QCA and computational fluid dynamics (Medis, CathWorks, and Philips) [104]. It is unclear if this technology is reliable in LM lesions, as in the setting of the LM a reference vessel is lacking. More promising for LM lesion evaluation is the technology to estimate FFR on angiographic images obtained with a computer tomography (CT) scan (FFR_CT_). CT enables a thorough assessment of the vascular wall, plaque distribution, and evaluation of the subtended myocardium. Using refined algorithms and computational fluid dynamics, coronary flow and pressure are computed under conditions simulating maximal hyperemia. FFR_CT_ values correlate strongly with invasively derived FFR, although FFR_CT_ values are systematically lower than invasive values and the algorithms assume a normal microcirculatory function [105]. Systematic data in LM lesions are lacking for now, but FFR_CT_ may become a useful tool in this setting, both for advanced screening purposes and for follow-up of lesions initially deferred for treatment and maybe even for follow-up of LM stent results.

#### 3.4.3. IVUS

Intravascular imaging techniques and in particular IVUS assess the anatomical severity of a lesion more accurately, being able to look for the true vessel diameter and plaque burden. IVUS has received a Class IIa indication in the latest ESC guidelines to assess the severity of the LM lesion [4]. Although coronary flow and pressure are not only determined by the anatomical severity of a stenosis, FFR has been shown to correlate better to the IVUS-derived minimal lumen area (MLA) in LM lesions than in non-LM lesions [20, 106]. This may be because the LM has a limitedly variable length and large diameter [20, 106]. Cut-offs for predicting physiological significance have been proposed ranging from 4.5 mm^2^ up to 5.9 mm^2^ [13]. The smaller cut-offs originated from studies in Asian patients, which usually had smaller LM MLAs overall [13]. The fact that in one study an FFR <0.80 was found in 24% of patients with an MLA >4.5 mm^2^ (the lowest cut-off) and that in another study an FFR >0.80 was found in 36% of patients with an MLA <6 mm^2^ (the highest cut-off) highlights the difficulty with IVUS as a rule-in tool for revascularization [13]. Strong evidence from the LITRO study suggests however that an MLA >6 mm^2^ can safely be used to defer revascularization, as 3-year cardiac death-free survival was 97.7% in those patients [107]. IVUS experts suggest deferring LM lesions with an MLA >6 mm^2^, proving functional significance by other means (e.g., invasive physiological assessment) when the MLA is between 5 and 6 mm^2^, and treating LM lesions with an MLA <5 mm^2^ [13].

Although physiological assessment of LM lesion severity received a higher recommendation than IVUS in the guidelines, IVUS should certainly be considered if physiological examination of the LM is for some reason difficult, impossible, or less reliable as highlighted above [4]. MLA assessment of a LM lesion is just a fraction of the potential of IVUS in LM. It also allows assessment of plaque burden (PB) and composition, vessel size and remodeling, and thorough assessment of the bifurcation. The finding of adverse plaque characteristics is relevant as it likely affects prognosis, as in FFR-deferred non-LM lesions [108]. However, a real strength of IVUS lies in the pre, per, and postprocedural guidance of LM PCI, which often involves the bifurcation [4]. Evidence from EXCEL and NOBLE, among other studies, indicates that appropriate IVUS guidance can lead to better stent results, with bigger final minimal stent areas (MSA) and better outcome [108, 109]. If the possibility of PCI is considered, one should have a very low threshold to assess it upfront with IVUS as well [13]. An illustrative case of a LM lesion with downstream disease assessed with both physiology and imaging is presented in Figure 1.

## 4. Limitations of Physiology as a Gatekeeper

Plaque rupture or thrombotic events, but also inappropriate revascularization, may have more devastating consequences when it concerns a LM lesion, in comparison to a lesion further down the coronary tree [21]. One should therefore consider the limitations of physiological assessment as a method to predict outcome.

### 4.1. Physiology and Outcome

There is evidence that links physiological findings to coronary events [16]. The FAME 2 study showed that PCI of lesions with an FFR ≤0.80 lowers MACE substantially up to 5 years follow-up (13.9% vs. 27.0% in the MT group; HR: 0.46; 95% CI: 0.34 to 0.63; *P* < 0.001) [110]. This was mainly driven by a significant reduction in urgent revascularizations in the PCI group. Importantly, meanwhile, a patient-level meta-analysis of all the available randomized trials of FFR-guided PCI vs. MT (involving 2400 subjects in total), now powered for the prespecified composite endpoint of cardiac death and MI, showed for the first time that FFR-guided PCI can reduce cardiac death or MI, by 28% (HR: 0.72; 95% CI: 0.54–0.96; *P*=0.024) after a mean follow-up of 35 months. This corresponds to an absolute risk reduction of 5.7% at 5 years and a number-needed-to-treat (NNT) of 18 [32]. For iFR, very long-term data are awaited to confirm that an iFR-based strategy can indeed reduce the need for MR while retaining similar outcomes as with an FFR-based strategy.

### 4.2. Ischemia and Outcome

There is evidence indicating that ischemia is a predictor of MACE and that revascularization improves survival over MT once ischemia extends to more than 10–12% of the myocardium, with treatment resulting in ≥5% ischemia reduction [7, 9, 16]. Analysis of patient-level FFR values and outcome indicates that FFR demonstrates a continuous instead of a binary relationship with outcome [111]. The lower the FFR, the higher the MACE rate, and the larger the benefit of revascularization over MT [111]. A lower pre-PCI FFR or iFR, also predicts more improvement of stress echocardiography parameters after PCI [112]. The optimal FFR threshold in favor of revascularization over MT was found to be ≤0.67 [111, 113]. These findings, together with data suggesting a better correlation of lower thresholds with alternative indices of ischemia and data suggesting that treatment of lesions with an FFR between 0.75 and 0.80 (the “grey zone”) is not associated with improved long-term outcomes, make some authors suggest more rigorous treatment thresholds for FFR and iFR [94–96]. Outcome data for such an approach are however lacking, and therefore experts argue for using the validated cut-offs [114]. They do suggest that borderline lesions may be deferred if the clinical situation suggests so (for example, minimal symptoms or high PCI risk) and expect that assessment of the microcirculation may become more important to predict potential benefit of PCI [95, 115]. Recently, the results of the ISCHEMIA trial were reported [5]. 5179 patients with moderate or severe ischemia were randomized to MT alone or MT and an invasive strategy ideally followed by optimal MR. Over a median follow-up of 3.3 years, the composite primary endpoint of cardiovascular death, MI, resuscitated cardiac death, or hospitalization for unstable angina or heart failure was not significantly different between both groups (15.5% vs. 13.3%; HR: 0.93; CI: 0.80 to 1.08; *P*=0.34). However, the outcome curves crossed after about 2 years, with an initial 1.9% cumulative disadvantage for the invasive strategy (attributable to more procedural MIs occurring in the invasive arm) turning into a 2.2% advantage (attributable to fewer nonprocedural spontaneous MIs occurring in the invasive arm). Within the relatively short follow-up, this did not result in a difference in the primary endpoint, but it might suggest a possible prognostic benefit with MR in the long run. Importantly, patients in the invasive arm had significant, durable improvements in angina control and quality of life with an invasive strategy provided that they had angina. One should point out that patients with severe angina and patients with LM disease were excluded from this trial and that 23% of the MT patients got MR during follow-up.

### 4.3. Plaque Vulnerability

There is likely also a relationship between lesion severity and plaque vulnerability, as high stenosis grades are found at the time of MI and high plaque burden and small MLA are predictive of future events [16, 116]. When analyzing previously performed coronary CTs with FFR_CT_ technology, one found that culprit lesions had lower FFR and higher delta FFR, wall shear stress, and axial plaque stress in comparison to nonculprit lesions [117]. It is hypothesized that the ischemic potential of a lesion, as assessed by physiology, can therefore be a marker of plaque vulnerability [16]. An obstructive, ischemia-producing lesion is thought to be more prone to cause an ACS than a non-ischemia-producing lesion, because in the former plaque progression will more likely lead to vessel occlusion and because increasing stenosis severity affects flow dynamics and wall shear stress making plaque rupture more likely [16]. The presence of a pressure gradient at the epicardial lesion level on its own (without necessarily ischemic potential at myocardial level) also appears to negatively impact prognosis. Data from DEFINE-FLOW indicate that patients with an FFR+lesion with preserved CFR (often found in iFR-/FFR+patients) treated medically had higher adverse event rates at 2 years than patients with a FFR- lesion and preserved CFR (10.8% vs. 5.8%; 5.0% difference; 95% CI: −1.5%–11.5%; *P* value for noninferiority: 0.065) [74, 83–85, 118].

Because of the higher flow passing through the LM and because of the large bifurcation it involves, the flow dynamics and plaque characteristics are particularly complex [119].

One should consider that refraining from revascularization of physiologically significant lesions does not always result in future clinical events. The fact that, in FAME 2 during the 5-year follow-up, MACE occurred in 27% of the medically treated patients with an FFR+lesion (vs. 13.9% in PCI group) also points out that 73% of those patients remained event free. Conversely, clinical events can still occur after deferring revascularization of lesions that are considered physiologically insignificant. The 5-year MACE rate of the patients with a deferred FFR- lesion included in the FAME 2 registry was as high as 15.7% [110]. Patients in DEFINE-FLOW with an FFR- lesion but impaired CFR (often found in iFR+/FFR- patients) also had bad outcomes (12.4% MACE at 2 years) [74, 83–85, 118].

Of note, the initially more pronounced angina relief with PCI than with MT in FAME 2 was no longer significant at 5 years [110]. This may be because 51% of the patients who had been assigned to MT by then had crossed over to PCI, but there is likely also some placebo effect of PCI, as suggested in the sham-controlled ORBITA trial [120]. Although complete freedom of angina was more frequent with PCI (NNT of 5) and ischemia as assessed by dobutamine stress echocardiography was improved (and more so in case of a lower FFR or iFR), angina was overall not less frequent compared to optimized MT alone in ORBITA [120, 121]. There was also no interaction between FFR or iFR and the effect of PCI on angina [120, 121].

Other factors, like plaque and patient characteristics, may not be thoroughly assessable with physiological assessment alone but likely play a pivotal role in plaque rupture and other cardiac events. In the landmark PROSPECT trial, future cardiovascular events after an ACS were equally attributable to a recurrent event at the site of former culprit lesion as to a former nonculprit lesion and the latter were initially mostly angiographically nonsignificant (DS < 30% in 30.2%; DS < 50% in 67.0%). Most of the nonculprit lesions responsible for future events were still thin-cap fibroatheroma (TCFA) (as identified on IVUS with virtual histology) or had an MLA ≤4.0 mm^2^, a PB ≥ 70%, or a combination of these characteristics [116]. The presence of TCFA was also a strong predictor of future MACE in diabetic patients with FFR- lesions included in the COMBINE study (HR: 4.60; 95% CI: 1.95–10.96; *P* < 0.001) (data presented by Elvin Kedhi at TCT connect 2020) [122]. In the PROSPECT II trial, the majority of events occurring after an ACS could be related to untreated non-flow-limiting nonculprit lesions, and PB ≥ 70% and a high lipid core burden (as determined by near-infrared spectroscopy, NIRS) were even more predictive than MLA for lesion-level MACE (data presented by David Erlinge at TCT connect 2020) [123]. FFR does correlate with adverse plaque characteristics assessed by coronary, and better so than iFR according to a recent report [124–127].

The presence of insulin-dependent diabetes, as a patient characteristic, was also one of the strongest independent predictors of future events in PROSPECT [116]. In DEFINE-FLAIR, MACE was significantly higher at one year in diabetics than in nondiabetics, with a comparable risk of MACE in both iFR- and FFR-guided treatment groups [128]. In iFR-SWEDEHEART, MACE was higher at 2 years in the diabetic patients evaluated with FFR vs. those evaluated with iFR, as presented by Ole Fröbert at TCT 2018. In the second-largest registry on FFR in LM, diabetes was an independent predictor of MACE in patients with a LM lesion evaluated as nonsignificant by FFR [41]. The investigators suggested more burden of disease and aggressive progression in diabetics, but also a possible underestimation of the LM lesion by FFR due to microvascular disease [41].

Only assessing the physiological impact of a LM lesion will thus always be an oversimplification of the disease complexity. In deciding upon an individual treatment, one should therefore take into account all relevant information, like patient demography, bystander coronary artery disease, symptoms, and evidence from noninvasive testing [21]. It is not unthinkable that future trials may indicate that patients with nonhemodynamically significant LM disease, but vulnerable morphological characteristics, may also benefit from an intervention [21].

## 5. Conclusion

**Figure 2:**
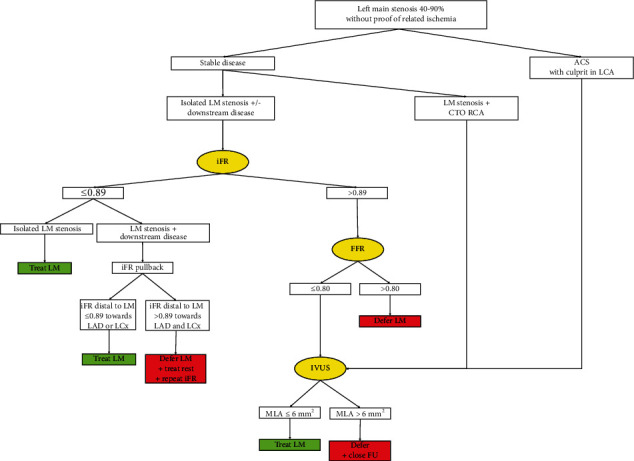
Our approach for intermediate LM lesions. Current approach to intermediate left main lesions in our center. The authors have a low threshold to use IVUS at any stage in the evaluation and treatment of LM lesions because of its proven added value as explained in the text. LM: left main; iFR: instantaneous wave-free ratio; FFR: fractional flow reserve; IVUS: intravascular ultrasound; ACS: acute coronary syndrome; CTO: chronic total occlusion; MLA: minimal lumen area; FU: follow-up.

Coronary angiography falls short of the assessment of intermediate LM lesions. Current guidelines consider a physiologic assessment by FFR or iFR the best way to assess the functional significance of a LM lesion. Most evidence supports FFR, but the studies were only observational, and only one used the current 0.80 cut-off. The best approach for LM lesions with an FFR of 0.75–≤0.80 remains somewhat debatable. iFR is promising since downstream disease is common in patients with LM disease and FFR is more difficult to interpret in the setting of serial lesions. The outcome of an iFR-based revascularization strategy has been shown to be noninferior to an FFR-based strategy, but in the setting of a LM lesion, data are limited to one observational study. Several studies are ongoing, like the iLITRO and our own multicenter PHYNAL registry (Prospective Left Main Physiology Registry), but only a randomized trial can confirm noninferiority of an iFR-based revascularization strategy. When confronted with discordant results of iFR and FFR, following one result over the other remains controversial but there are no clear indications that following the iFR result in such a case is inferior. Although physiology aids in the decision making with regard to revascularization, one needs to stay aware that the natural history of coronary atherosclerotic lesions is complex and physiological assessment with FFR or iFR cannot completely predict outcome, whether MR is performed or not. Therefore, if one decides to defer a LM lesion, close follow-up is recommended. Given the current evidence and guidelines presented above, we present our approach when confronted with an intermediate LM lesion, without prior proof of related ischemia (Figure 2).

## Figures and Tables

**Table 1:** LM revascularization decision making based on FFR or iFR: observational outcome studies.

	Cut-off	Total number of patients	Total number of deferred patients (physiologically nonsignificant)	Total number of revascularized patients (physiologically significant)	Mean follow-up (months)	Odds ratio for MACE of deferred vs. revascularized [36]
*FFR in LM*						
Bech et al. 2001 [37]	0.75	54	24	30	29 ± 15	1.316 (*P*=0.696)
Jiménez-Navarro et al. 2004 [38]	0.75	27	20	7	26 ± 12	0.625 (*P*=0.640)
Legutko et al. 2005 [39]	0.75	38	20	18	24 ± 12	0.889 (*P*=0.911)
Lindstaedt et al. 2006 [40]	0.75^*∗*^	51	24	27	29 ± 16	0.952 (*P*=0.940)
Courtis et al. 2009 [41]	0.75^*∗*^	142	82	60	14 ± 11	3.394 (*P*=0.038)
Hamilos et al. 2009 [42]	0.80	213	138	75	36 (6–99)	1.415 (*P*=0.374)
Total		**525**	308	217		**1.434**(*P*=0.152)

*iFR in LM*						
Warisawa et al. 2020 [43]	0.89	314	163	151	30	1.45 (*P*=0.26)
*Total*		**314**	163	151		**1.45**(*P*=0.26)

LM: left main; FFR: fractional flow reserve; iFR: instantaneous wave-free ratio; ^*∗*^for FFR between 0.75 and 0.80, additional clinical data were used to proceed with revascularization.

**Table 2:** Main strengths and weaknesses of FFR and iFR in LM revascularization decision making.

	Strengths	Weaknesses
FFR	(1) Strong long-term evidence from RCTs and meta-analyses in non-LM lesions (2) Evidence from observational studies and meta-analysis in >500 LM patients	(1) Nonuniform threshold in LM studies (2) Limited evidence for MR if FFR in grey zone (3) Influence of major daughter branch or distal disease (4) Influence of microcirculatory response to a hyperemic stimulus

iFR	(1) Strong medium-term evidence from RCTs in non-LM patients (2) Less influence expected of major daughter branch or distal disease	(1) Limited evidence from 1 observational study in LM patients

LM: left main; FFR: fractional flow reserve; iFR: instantaneous wave-free ratio; RCT: randomized controlled trial; MR: myocardial revascularization.

## References

[1] Kolh P., Windecker S., Alfonso F. (2014). 2014 ESC/EACTS guidelines on myocardial revascularization. *European Journal of Cardio-thoracic Surgery*.

[2] Taylor H. A., Deumite N. J., Chaitman B. R., Davis K. B., Killip T., Rogers W. J. (1989). Asymptomatic left main coronary artery disease in the coronary artery surgery study (CASS) registry. *Circulation*.

[3] Yusuf S., Zucker D., Passamani E. (1994). Effect of coronary artery bypass graft surgery on survival: overview of 10-year results from randomised trials by the coronary artery bypass graft surgery trialists collaboration. *The Lancet*.

[4] Neumann F.-J., Sousa-Uva M., Ahlsson A. (2018). 2018 ESC/EACTS guidelines on myocardial revascularization. *European Heart Journal*.

[5] Maron D. J., Hochman J. S., Reynolds H. R. (2020). Initial invasive or conservative strategy for stable coronary disease. *The New England Journal of Medicine*.

[6] Boden W. E., O’Rourke R. A., Teo K. K. (2007). Optimal medical therapy with or without PCI for stable coronary disease. *New England Journal of Medicine*.

[7] Hachamovitch R., Hayes S. W., Friedman J. D., Cohen I., Berman D. S. (2003). Comparison of the short-term survival benefit associated with revascularization compared with medical therapy in patients with no prior coronary artery disease undergoing stress myocardial perfusion single photon emission computed tomography. *Circulation*.

[8] Hachamovitch R., Rozanski A., Shaw L. J. (2011). Impact of ischaemia and scar on the therapeutic benefit derived from myocardial revascularization vs. medical therapy among patients undergoing stress-rest myocardial perfusion scintigraphy. *European Heart Journal*.

[9] Shaw L. J., Berman D. S., Maron D. J. (2008). Optimal medical therapy with or without percutaneous coronary intervention to reduce ischemic burden. *Circulation*.

[10] Leaman D. M., Brower R. W., Meester G. T., Serruys P., van den Brand M. (1981). Coronary artery atherosclerosis: severity of the disease, severity of angina pectoris and compromised left ventricular function. *Circulation*.

[11] Cerci R. J., Arbab-Zadeh A., George R. T. (2012). Aligning coronary anatomy and myocardial perfusion territories. *Circulation: Cardiovascular Imaging*.

[12] Stone G. W., Kappetein A. P., Sabik J. F. (2019). Five-year outcomes after PCI or CABG for left main coronary disease. *New England Journal of Medicine*.

[13] Kassimis G., de Maria G. L., Patel N. (2018). Assessing the left main stem in the cardiac catheterization laboratory. What is “significant”? Function, imaging or both?. *Cardiovascular Revascularization Medicine*.

[14] Stone G. W., Sabik J. F., Serruys P. W. (2016). Everolimus-eluting stents or bypass surgery for left main coronary artery disease. *New England Journal of Medicine*.

[15] Mäkikallio T., Holm N. R., Lindsay M. (2016). Percutaneous coronary angioplasty versus coronary artery bypass grafting in treatment of unprotected left main stenosis (NOBLE): a prospective, randomised, open-label, non-inferiority trial. *The Lancet*.

[16] Ford T. J., Berry C., De Bruyne B. (2017). Physiological predictors of acute coronary syndromes. *JACC: Cardiovascular Interventions*.

[17] Kan J., Gao X., Sandeep K. G. (2014). Comparison of two and three dimensional quantitative coronary angiography to intravascular ultrasound in the assessment of left main coronary artery bifurcation lesions. *Chinese Medical Journal*.

[18] Rab T., Sheiban I., Louvard Y., Sawaya F. J., Zhang J. J., Chen S. L. (2017). Current interventions for the left main bifurcation. *JACC: Cardiovascular Interventions*.

[19] Kern M. J., Samady H. (2010). Current concepts of integrated coronary physiology in the catheterization laboratory. *Journal of the American College of Cardiology*.

[20] Bhavik M., Vdht P., Jan P. J., Divaka P. (2017). Physiological assessment of left main coronary artery disease. *Eurointervention*.

[21] Stone G. W. (2020). Deferred revascularization of intermediate left main lesions. *JACC: Cardiovascular Interventions*.

[22] Fisher L. D., Judkins M. P., Lesperance J. (1982). Reproducibility of coronary arteriographic reading in the coronary artery surgery study (CASS). *Catheterization and Cardiovascular Diagnosis*.

[23] Isner J. M., Kishel J., Kent K. M., Ronan J. A., Ross A. M., Roberts W. C. (1981). Accuracy of angiographic determination of left main coronary arterial narrowing. angiographic—histologic correlative analysis in 28 patients. *Circulation*.

[24] Shiono Y., Kubo T., Tanaka A. (2014). Impact of myocardial supply area on the transstenotic hemodynamics as determined by fractional flow reserve. *Catheterization and Cardiovascular Interventions*.

[25] Kim H. Y., Lim H.-S., Doh J.-H. (2016). Physiological severity of coronary artery stenosis depends on the amount of myocardial mass subtended by the coronary artery. *JACC: Cardiovascular Interventions*.

[26] van de Hoef T. P., Meuwissen M., Escaned J. (2013). Fractional flow reserve as a surrogate for inducible myocardial ischaemia. *Nature Reviews Cardiology*.

[27] Modi B. N., De Silva K., Rajani R., Curzen N., Perera D. (2018). Physiology-guided management of serial coronary artery disease. *JAMA Cardiology*.

[28] Leone A. M., De Caterina A. R., Basile E. (2013). Influence of the amount of myocardium subtended by a stenosis on fractional flow reserve. *Circulation: Cardiovascular Interventions*.

[29] Van De Hoef T. P., Siebes M., Spaan J. A. E., Piek J. J. (2015). Fundamentals in clinical coronary physiology: why coronary flow is more important than coronary pressure. *European Heart Journal*.

[30] Toth G., Hamilos M., Pyxaras S. (2014). Evolving concepts of angiogram: fractional flow reserve discordances in 4000 coronary stenoses. *European Heart Journal*.

[31] Pijls N. H., van Son J. A., Kirkeeide R. L., De Bruyne B., Gould K. L. (1993). Experimental basis of determining maximum coronary, myocardial, and collateral blood flow by pressure measurements for assessing functional stenosis severity before and after percutaneous transluminal coronary angioplasty. *Circulation*.

[32] Zimmermann F. M., Omerovic E., Fournier S. (2019). Fractional flow reserve-guided percutaneous coronary intervention vs. medical therapy for patients with stable coronary lesions: meta-analysis of individual patient data. *European Heart Journal*.

[33] Tonino P. A. L., De Bruyne B., Pijls N. H. J. (2009). Fractional flow reserve versus angiography for guiding percutaneous coronary intervention. *New England Journal of Medicine*.

[34] De Bruyne B., Fearon W. F., Pijls N. H. J. (2014). Fractional flow reserve-guided PCI for stable coronary artery disease. *New England Journal of Medicine*.

[35] Bech G. J. W., De Bruyne B., Pijls N. H. J. (2001). Fractional flow reserve to determine the appropriateness of angioplasty in moderate coronary stenosis. *Circulation*.

[36] Mallidi J., Atreya A. R., Cook J. (2015). Long-term outcomes following fractional flow reserve-guided treatment of angiographically ambiguous left main coronary artery disease: a meta-analysis of prospective cohort studies. *Catheterization and Cardiovascular Interventions*.

[37] Bech G. J. W., Droste H., Pijls N. H. (2001). Value of fractional flow reserve in making decisions about bypass surgery for equivocal left main coronary artery disease. *Heart*.

[38] Jiménez-Navarro M., Hernández-García J. M., Alonso-Briales J. H. (2004). Should we treat patients with moderately severe stenosis of the left main coronary artery and negative FFR results?. *Journal of Invasive Cardiology*.

[39] Legutko J., Dudek D., Rzeszutko L., Wizimirski M., Dubiel J. S. (2005). Fractional flow reserve assessment to determine the indications for myocardial revascularisation in patients with borderline stenosis of the left main coronary artery. *Kardiologia Polska*.

[40] Lindstaedt M., Yazar A., Germing A. (2006). Clinical outcome in patients with intermediate or equivocal left main coronary artery disease after deferral of surgical revascularization on the basis of fractional flow reserve measurements. *American Heart Journal*.

[41] Courtis J., Rodés-Cabau J., Larose E. (2009). Usefulness of coronary fractional flow reserve measurements in guiding clinical decisions in intermediate or equivocal left main coronary stenoses. *The American Journal of Cardiology*.

[42] Hamilos M., Muller O., Cuisset T. (2009). Long-term clinical outcome after fractional flow reserve-guided treatment in patients with angiographically equivocal left main coronary artery stenosis. *Circulation*.

[43] Warisawa T., Cook C. M., Rajkumar C. (2020). Safety of revascularization deferral of left main stenosis based on instantaneous wave-free ratio evaluation. *JACC: Cardiovascular Interventions*.

[44] Kuramitsu S., Matsuo H., Shinozaki T. (2020). Two-year outcomes after deferral of revascularization based on fractional flow reserve: the J-confirm registry. *Circulation: Cardiovascular Interventions*.

[45] Gould K. L., Lipscomb K., Hamilton G. W. (1974). Physiologic basis for assessing critical coronary stenosis. *The American Journal of Cardiology*.

[46] De Bruyne B., Pijls N. H. J., Heyndrickx G. R., Hodeige D., Kirkeeide R., Gould K. L. (2000). Pressure-derived fractional flow reserve to assess serial epicardial stenoses. *Circulation*.

[47] Pijls N. H. J., De Bruyne B., Bech G. J. W. (2000). Coronary pressure measurement to assess the hemodynamic significance of serial stenoses within one coronary artery. *Circulation*.

[48] Park S.-J., Ahn J.-M., Pijls N. H. J. (2012). Validation of functional state of coronary tandem lesions using computational flow dynamics. *The American Journal of Cardiology*.

[49] Kim H.-L., Koo B.-K., Nam C.-W. (2012). Clinical and physiological outcomes of fractional flow reserve-guided percutaneous coronary intervention in patients with serial stenoses within one coronary artery. *JACC: Cardiovascular Interventions*.

[50] Kern M. J., Seto A. H. (2018). A perspective on physiologic assessment of coronary stenoses in series. *JAMA Cardiology*.

[51] Nijjer S. S., Sen S., Petraco R., Mayet J., Francis D. P., Davies J. E. R. (2015). The instantaneous wave-free ratio (iFR) pullback: a novel innovation using baseline physiology to optimise coronary angioplasty in tandem lesions. *Cardiovascular Revascularization Medicine*.

[52] Jeremias A., Davies J. E., Maehara A. (2019). Blinded physiological assessment of residual ischemia after successful angiographic percutaneous coronary intervention. *JACC: Cardiovascular Interventions*.

[53] Yong A. S. C., Daniels D., De Bruyne B. (2013). Fractional flow reserve assessment of left main stenosis in the presence of downstream coronary stenoses. *Circulation: Cardiovascular Interventions*.

[54] Daniels D. V., van’t Veer M., Pijls N. H. J. (2012). The impact of downstream coronary stenoses on fractional flow reserve assessment of intermediate left main disease. *JACC: Cardiovascular Interventions*.

[55] Fearon W. F., Yong A. S., Lenders G. (2015). The impact of downstream coronary stenosis on fractional flow reserve assessment of intermediate left main coronary artery disease. *JACC: Cardiovascular Interventions*.

[56] Yamamoto E., Watanabe S., Saito N., Kawase Y., Kimura T. (2014). TCT-312 prediction of the true fractional flow reserve of left main coronary artery stenosis with concomitant downstream stenoses: in vitro and in vivo experiments. *Journal of the American College of Cardiology*.

[57] Kern M. J. (2016). My approach to the patient diagnosed with significant left main disease: use of FFR, IVUS, and OCT. *Trends in Cardiovascular Medicine*.

[58] Holmvang G., Fry S., Skopicki H. A. (1999). Relation between coronary “steal” and contractile function at rest in collateral-dependent myocardium of humans with ischemic heart disease. *Circulation*.

[59] Ladwiniec A., Cunnington M. S., Rossington J. (2015). Collateral donor artery physiology and the influence of a chronic total occlusion on fractional flow reserve. *Circulation: Cardiovascular Interventions*.

[60] Mohdnazri S. R., Karamasis G. V., Al-Janabi F. (2018). The impact of coronary chronic total occlusion percutaneous coronary intervention upon donor vessel fractional flow reserve and instantaneous wave-free ratio: implications for physiology-guided PCI in patients with CTO. *Catheterization and Cardiovascular Interventions*.

[61] Meuwissen M., Chamuleau S. A. J., Siebes M. (2001). Role of variability in microvascular resistance on fractional flow reserve and coronary blood flow velocity reserve in intermediate coronary lesions. *Circulation*.

[62] Layland J., Oldroyd K. G., Curzen N. (2015). Fractional flow reserve vs. angiography in guiding management to optimize outcomes in non-ST-segment elevation myocardial infarction: the British Heart Foundation FAMOUS-NSTEMI randomized trial. *European Heart Journal*.

[63] Cuculi F., De Maria G. L., Meier P. (2014). Impact of microvascular obstruction on the assessment of coronary flow reserve, index of microcirculatory resistance, and fractional flow reserve after ST-segment elevation myocardial infarction. *Journal of the American College of Cardiology*.

[64] Escaned J., Ryan N., Mejía-Rentería H. (2018). Safety of the deferral of coronary revascularization on the basis of instantaneous wave-free ratio and fractional flow reserve measurements in stable coronary artery disease and acute coronary syndromes. *JACC: Cardiovascular Interventions*.

[65] Cerrato E., Mejía-Rentería H., Dehbi H.-M. (2020). Revascularization deferral of nonculprit stenoses on the basis of fractional flow reserve. *JACC: Cardiovascular Interventions*.

[66] Toth G. G., De Bruyne B., Rusinaru D. (2016). Impact of right atrial pressure on fractional flow reserve measurements. *JACC: Cardiovascular Interventions*.

[67] Cook C., Ahmad Y., Petraco R. (2015). TCT-42 accounting for right atrial pressure in the calculation of fractional flow reserve (FFR) significantly increases the number of physiologically significant stenoses suitable for PCI. *Journal of the American College of Cardiology*.

[68] Perera D., Biggart S., Postema P. (2004). Right atrial pressure: can it be ignored when calculating fractional flow reserve and collateral flow index?. *Journal of the American College of Cardiology*.

[69] Sen S., Escaned J., Malik I. S. (2012). Development and validation of a new adenosine-independent index of stenosis severity from coronary wave-intensity analysis. *Journal of the American College of Cardiology*.

[70] Nijjer S. S., de Waard G. A., Sen S. (2016). Coronary pressure and flow relationships in humans: phasic analysis of normal and pathological vessels and the implications for stenosis assessment: a report from the Iberian-Dutch-English (IDEAL) collaborators. *European Heart Journal*.

[71] Götberg M., Cook C. M., Sen S., Nijjer S., Escaned J., Davies J. E. (2017). The evolving future of instantaneous wave-free ratio and fractional flow reserve. *Journal of the American College of Cardiology*.

[72] van de Hoef T. P., Lee J. M., Echavarria-Pinto M. (2020). Non-hyperaemic coronary pressure measurements to guide coronary interventions. *Nature Reviews Cardiology*.

[73] Hwang D., Jeon K.-H., Lee J. M. (2017). Diagnostic performance of resting and hyperemic invasive physiological indices to define myocardial ischemia. *JACC: Cardiovascular Interventions*.

[74] Petraco R., Van De Hoef T. P., Nijjer S. (2014). Baseline instantaneous wave-free ratio as a pressure-only estimation of underlying coronary flow reserve. *Circulation: Cardiovascular Interventions*.

[75] Davies J. E., Sen S., Dehbi H. M. (2017). Use of the instantaneous wave-free ratio or fractional flow reserve in PCI. *The New England Journal of Medicine*.

[76] Götberg M., Christiansen E. H., Gudmundsdottir I. J. (2017). Instantaneous wave-free ratio versus fractional flow reserve to guide PCI. *New England Journal of Medicine*.

[77] Cost-effectiveness of instantaneous wave-Free Ratio (iFR) compared with Fractional Flow Reserve (FFR) to guide coronary revascularization decision-making–analysis from DEFINE FLAIR. Presented by Allen Jeremias at the 2018 ACC Meeting

[78] Cook C. M., Warisawa T., Howard J. P. (2019). Algorithmic versus expert human interpretation of instantaneous wave-free ratio coronary pressure-wire pull back data. *JACC: Cardiovascular Interventions*.

[79] Nijjer S. S., Sen S., Petraco R. (2014). Pre-angioplasty instantaneous wave-free ratio pullback provides virtual intervention and predicts hemodynamic outcome for serial lesions and diffuse coronary artery disease. *JACC: Cardiovascular Interventions*.

[80] Kikuta Y., Cook C. M., Sharp A. S. P. (2018). Pre-angioplasty instantaneous wave-free ratio pullback predicts hemodynamic outcome in humans with coronary artery disease. *JACC: Cardiovascular Interventions*.

[81] Frimerman A., Abu-Fane R., Levi Y. (2019). Novel method for real-time coregistration of coronary physiology and angiography by iFR. *JACC: Cardiovascular Interventions*.

[82] Rosa P., Velli C., Sorrentino S. (2019). Reliability of instantaneous wave-free ratio (iFR) for the evaluation of left main coronary artery lesions. *Journal of Clinical Medicine*.

[83] Lee J. M., Hwang D., Park J., Tong Y., Koo B.-K. (2017). Physiologic mechanism of discordance between instantaneous wave-free ratio and fractional flow reserve: insight from 13 N-ammonium positron emission tomography. *International Journal of Cardiology*.

[84] Stegehuis V. E., van de Hoef T. P., Piek J. J., Claessen B. E. (2018). Go with the flow when instantaneous wave-free ratio-fractional flow reserve discordance occurs. *JACC: Cardiovascular Interventions*.

[85] Cook C. M., Jeremias A., Petraco R. (2017). Fractional flow reserve/instantaneous wave-free ratio discordance in angiographically intermediate coronary stenoses. *JACC: Cardiovascular Interventions*.

[86] Kobayashi Y., Johnson N. P., Berry C. (2016). The influence of lesion location on the diagnostic accuracy of adenosine-free coronary pressure wire measurements. *JACC: Cardiovascular Interventions*.

[87] Jeremias A., Maehara A., Généreux P. (2014). Multicenter core laboratory comparison of the instantaneous wave-free ratio and resting P/P with fractional flow reserve. *Journal of the American College of Cardiology*.

[88] Dérimay F., Johnson N. P., Zimmermann F. M. (2019). Predictive factors of discordance between the instantaneous wave-free ratio and fractional flow reserve. *Catheterization and Cardiovascular Interventions*.

[89] Lee J. M., Shin E.-S., Nam C.-W. (2017). Discrepancy between fractional flow reserve and instantaneous wave-free ratio: clinical and angiographic characteristics. *International Journal of Cardiology*.

[90] Kirigaya H., Matsushita K., Okada K. (2018). TCT-152 clinical predictors of discordance between instantaneous wave-free ratio and fractional flow reserve. *Journal of the American College of Cardiology*.

[91] van de Hoef T. P., Echavarria-Pinto M., Meuwissen M., Stegehuis V. E., Escaned J., Piek J. J. (2020). Contribution of age-related microvascular dysfunction to abnormal coronary. *JACC: Cardiovascular Interventions*.

[92] Two-year outcomes of patients with revascularisation deferral based on FFR or iFR measurements. Presented by Javier Escaned during the 2020 PCR e-Course

[93] Lee J. M., Shin E.-S., Nam C.-W. (2017). Clinical outcomes according to fractional flow reserve or instantaneous wave-free ratio in deferred lesions. *JACC: Cardiovascular Interventions*.

[94] Kang D.-Y., Ahn J.-M., Lee C. H. (2018). Deferred vs. performed revascularization for coronary stenosis with grey-zone fractional flow reserve values: data from the IRIS-FFR registry. *European Heart Journal*.

[95] Kern M. J., Seto A. H. (2018). Caution! You’re approaching a gray zone: FFR outcomes and the role of CFR and IMR. *Catheterization and Cardiovascular Interventions*.

[96] Hennigan B., Berry C., Collison D. (2020). Percutaneous coronary intervention versus medical therapy in patients with angina and grey-zone fractional flow reserve values: a randomised clinical trial. *Heart*.

[97] Sen S., Ahmad Y., Dehbi H.-M. (2019). Clinical events after deferral of LAD revascularization following physiological coronary assessment. *Journal of the American College of Cardiology*.

[98] Escaned J., Davies J. (2017). *Physiological Assessment of Coronary Stenoses and the Microcirculation*.

[99] Cook C. M., Ahmad Y., Shun-Shin M. J. (2016). Quantification of the effect of pressure wire drift on the diagnostic performance of fractional flow reserve, instantaneous wave-free ratio, and whole-cycle Pd/Pa. *Circulation: Cardiovascular Interventions*.

[100] Svanerud J., Ahn J.-M., Jeremias A. (2018). Validation of a novel non-hyperaemic index of coronary artery stenosis severity: the resting full-cycle ratio (validate RFR) study. *EuroIntervention*.

[101] Kern M. J., Berry C., deBruyne B. (2019). Conversation in cardiology: is there a need for clinical trials for the nonhyperemic pressure ratios?. *Catheterization and Cardiovascular Interventions*.

[102] IRIS-FFR Registry: Prognostic Performance of Five Resting Pressure- Derived Indexes of Coronary Physiology Disclosure. Presented by Jung-Min Ahn at the 2018 TCT meeting

[103] Lee J. M., Choi K. H., Park J. (2018). Physiologic and clinical assessment of resting physiologic Indices : resting full-cycle ratio, diastolic pressure-ratio, and instantaneous wave-free ratio. *Circulation*.

[104] Collet C., Onuma Y., Sonck J. (2018). Diagnostic performance of angiography-derived fractional flow reserve: a systematic review and Bayesian meta-analysis. *European Heart Journal*.

[105] Driessen R. S., Danad I., Stuijfzand W. J. (2019). Comparison of coronary computed tomography angiography, fractional flow reserve, and perfusion imaging for ischemia diagnosis. *Journal of the American College of Cardiology*.

[106] Nascimento B. R., de Sousa M. R., Koo B.-K. (2014). Diagnostic accuracy of intravascular ultrasound-derived minimal lumen area compared with fractional flow reserve-meta-analysis: pooled accuracy of IVUS luminal area versus FFR. *Catheterization and Cardiovascular Interventions*.

[107] de la Torre Hernandez J. M., Hernández Hernandez F., Alfonso F. (2011). Prospective application of pre-defined intravascular ultrasound criteria for assessment of intermediate left main coronary artery lesions. *Journal of the American College of Cardiology*.

[108] Maehara A., Mintz G. S., Stone G. W. (2020). IVUS guidance during left main PCI: not if, but when and how. *EuroIntervention*.

[109] Ladwiniec A., Walsh S. J., Holm N. R. (2020). Intravascular ultrasound to guide left main stem intervention: a NOBLE trial substudy. *EuroIntervention*.

[110] Xaplanteris P., Fournier S., Pijls N. H. J. (2018). Five-year outcomes with PCI guided by fractional flow reserve. *New England Journal of Medicine*.

[111] Johnson N. P., Tóth G. G., Lai D. (2014). Prognostic value of fractional flow reserve. *Journal of the American College of Cardiology*.

[112] Modi B. N., Rahman H., Kaier T. (2018). Revisiting the optimal fractional flow reserve and instantaneous wave-free ratio thresholds for predicting the physiological significance of coronary artery disease. *Circulation: Cardiovascular Interventions*.

[113] Barbato E., Toth G. G., Johnson N. P. (2016). A prospective natural history study of coronary atherosclerosis using fractional flow reserve. *Journal of the American College of Cardiology*.

[114] Davies J. E., Cook C. M. (2018). Is now the time to debate traditional fractional flow reserve/instantaneous wave-free ratio cut points?. *Circulation: Cardiovascular Interventions*.

[115] Niida T., Murai T., Yonetsu T. (2018). Coronary physiological assessment combining fractional flow reserve and index of microcirculatory resistance in patients undergoing elective percutaneous coronary intervention with grey zone fractional flow reserve. *Catheterization and Cardiovascular Interventions*.

[116] Stone G. W., Maehara A., Lansky A. J. (2011). A prospective natural-history study of coronary atherosclerosis. *New England Journal of Medicine*.

[117] Lee J. M., Choi G., Koo B. K. (2018). Identification of high-risk plaques destined to cause acute coronary syndrome using coronary computed tomographic angiography and computational fluid dynamics. *JACC Cardiovascular Imaging*.

[118] The DEFINE-FLOW study: combined CFR and FFR assessment. Presented by Nils Johnson during the 2020 TCT Connect Meeting

[119] Collet C., Capodanno D., Onuma Y. (2018). Left main coronary artery disease: pathophysiology, diagnosis, and treatment. *Nature Reviews Cardiology*.

[120] Al-Lamee R., Thompson D., Dehbi H. M. (2018). Percutaneous coronary intervention in stable angina (ORBITA): a double-blind, randomised controlled trial. *Lancet*.

[121] Al-Lamee R., Howard J. P., Shun-Shin M. J. (2018). Fractional flow reserve and instantaneous wave-free ratio as predictors of the placebo-controlled response to percutaneous coronary intervention in stable single-vessel coronary artery disease. *Circulation*.

[122] COMBINE (OCT-FFR): A prospective natural history study using OCT imaging in patients with diabetes. Presented by Elvin Kedhi during the 2020 TCT Connect Meeting

[123] PROSPECT II : A prospective natural history study using NIRS-IVUS imaging in patients with acute myocardial infarction. Presented by David Erlinge during the 2020 TCT Connect meeting

[124] Driessen R. S., de Waard G. A., Stuijfzand W. J. (2020). Adverse plaque characteristics relate more strongly with hyperemic fractional flow reserve and instantaneous wave-free ratio than with resting instantaneous wave-free ratio. *JACC: Cardiovascular Imaging*.

[125] Gaur S., Øvrehus K. A., Dey D. (2016). Coronary plaque quantification and fractional flow reserve by coronary computed tomography angiography identify ischaemia-causing lesions. *European Heart Journal*.

[126] Park H.-B., Heo R., ó Hartaigh B. (2015). Atherosclerotic plaque characteristics by CT angiography identify coronary lesions that cause ischemia. *JACC: Cardiovascular Imaging*.

[127] Driessen R. S., Stuijfzand W. J., Raijmakers P. G. (2018). Effect of plaque burden and morphology on myocardial blood flow and fractional flow reserve. *Journal of the American College of Cardiology*.

[128] Lee J. M., Choi K. H., Koo B.-K. (2019). Comparison of major adverse cardiac events between instantaneous wave-free ratio and fractional flow reserve-guided strategy in patients with or without type 2 diabetes: a secondary analysis of a randomized clinical trial. *JAMA Cardiology*.

